# Examining the Unconscious Racial Biases and Attitudes of Physicians, Nurses, and the Public: Implications for Future Health Care Education and Practice

**DOI:** 10.1089/heq.2021.0141

**Published:** 2022-05-18

**Authors:** Danielle D. Jones

**Affiliations:** American Academy of Family Physicians, Leawood, Kansas, USA.

**Keywords:** unconscious bias, health equity, health disparities, workforce

## Abstract

**Background:**

While studies have shown that unconscious bias (UB) is associated with racial health care disparities, its magnitude in the health care workforce has not been examined. Furthermore, there is an absence of studies examining the attitudes of health care workers toward UB, which may have implications for intervention effectiveness. This study aims to address these gaps to further understand the potential scope of impact of UB and interventions designed to address it on patient care.

**Methods:**

This study provides an analysis to understand the magnitude of UB among physicians and nurses and their attitudes. Comparisons are made to the public to infer the potential causes and influences of medical education and training on individuals' UB.

**Results:**

The health care workforce demonstrated a greater preference for whites than the public, nurses more so than physicians. UB was also shown to have significant geographic and professional variability. Nurses are more likely to agree that their UB is a reflection of the cultures they are exposed to unlike physicians who see their UB as an indication of individualistic or automatic thoughts toward people of another race.

**Conclusions:**

The UB of the health care workforce and their attitudes toward UB differ significantly from those of the general public. Current and future interventions aimed at reducing UB, to include education and policy changes, should consider these variations, especially when legislating mandates.

## Introduction

Studies have shown that despite the most egalitarian of viewpoints, bias is pervasive among all health care professions.^1^ A clinicians' ability to deliver a differential diagnosis and treatment that is both equitable and optimal is often limited by time, complexity and cognitive overload.^2,3^ However, the process may be further constrained by lack of cultural competency and/or unconscious bias (UB), especially when race is a factor, which has shown to increase racial health care disparities.^4–7^ For example, clinicians' racial biases have been associated with disparities related to premature death, pain management, coronary artery disease, kidney dialysis, contraception and prenatal care, as well as patient-provider communication, satisfaction, and adherence to treatment.^8–13^

In addition, clinicians' biases have been shown to also be moderated by their personal identity (i.e., race, gender).^14,15^ For example, in a study of implicit and explicit racial bias among medical doctors, black physicians showed no preference for either whites or blacks and females showed weaker preference for whites than males.

Much of the UB research has been generated using mostly primary care physicians as study participants.^16–18^ While some studies have included other types of clinicians and/or medical specialties, seldom if ever are those results stratified to allow for comparisons between groups. While the UB of physicians and providers as a whole have been thoroughly examined, little is known about the UB of nurses independent from other provider types. Wherein they are described in the literature, the focus is mostly didactic, only providing frameworks and strategies to mitigate the effects of UB in nursing education and practice.^19–24^

Advanced practice nurses are increasingly providing holistic patient centered care that requires them to make care decisions and treatment recommendations to prevent and manage complex biopsychosocial issues independent of physician oversight.^25–28^ These decisions are also subject to influence from UB, which justifies the need to examine nurses as thoroughly as physicians to infer their potential contribution to health care disparities.

This study aims to examine and distinguish the magnitude of UB and the attitudes of physicians and nurses toward UB. According to the primary care performance improvement literature, understanding the contextual factors of an intervention, such as individuals' attitudes toward it, is necessary as they are likely to moderate behaviors associated with effectiveness.^29,30^ Previous studies comparing the UB of primary care providers to the local community found no substantial differences and suggested bias should be considered more of a societal issue and less as a health care issue.^1^ If so, that would then suggest that even when stratified by type of provider, the UB of health care professionals are the same as those of the general public and shaped by the same social knowledge and experiences.

However, different outcomes for health care professionals would indicate that the UB of health care professionals are mediated by additional differential knowledge and experiences encountered throughout medical education, training, and practice, which may require alternative interventions.

## Measures and Methods

Data for this study come from Project Implicit, the most widely used and well-alidated measure of implicit associations, wherein respondents opt-in for their data to be used solely for the purpose of the scientific study of UB.^31^ Based upon data from the 2010 Census, Project Implicit respondents tend to be younger, female, and reflect the racial demographics of the regions, in which participants are located.^32^ This study examines data from a sample of 76,000 respondents to the self-identified occupation variable of the Race Implicit Association Test (IAT) from 2015 to 2019, which included nearly 3000 health care professionals, using Stata 15.1.

While the use of data from voluntary opt-in respondents may introduce some selection bias, it is not believed to skew any potential findings from this analysis and may in fact provide the basis for further research with a more clearly defined population.

Occupation data are available by 65 occupational categories, which include five categories for health care. As this study is specifically interested only in those health care occupations that provide diagnostic and treatment recommendations, the occupation variable was recoded to specify (1) medical doctors, (2) nurses, and (3) all other occupations. It is important to note that a limitation of this occupational data is that it does not specify the different types or levels of training among medical (i.e., MD vs. DO) and nursing (i.e., LPN, RN) respondents. There are differences in scope of practice between registered nurses and licensed practical nurses and education between BSN-prepared nurses and ADN-prepared nurses.

Likewise, the philosophy of care among Doctor of Osteopathic Medicine differs significantly from those trained in allopathic medicine. It is unclear at this time the potential impact these differences may have on their UB, but may present an opportunity for future research. Geographical locations are captured by state and recoded to one of six cultural regions based on aggregated attitudes and beliefs.^33^

Previous research has demonstrated that the UB of whites toward blacks aggregated at the county and state level are higher in the southeast and are also strongly correlated with disparities in mortality, birth outcomes, police brutality and Medicaid spending and vary by region.^34^ As suggested, if UB is a societal issue, then this regional variation should also be consistent among health care professionals and reflected in their perceptions overall.

The outcomes of interest in this study are the IAT D-score and attitudes. Unconscious associations are measured using a D score that has a theoretical range of −2 to +2.^35^ Respondents with a D-score equal to 0 (±0.15) demonstrate no preference for either white or black individuals, whereas more positive scores suggest a “slight” (0.15<), “moderate” (0.35<), or “strong” (0.65<) preference for whites.

To measure attitudes, survey participants were asked to reflect on the exercise using three statements to indicate their level of acceptance or disregard of their IAT results. It has been suggested that individuals who express agreement with and acceptance of these statements are able to quickly process and understand their negative unconscious associations and move toward actions that dismantle them.^33,36,37^ This may provide some insight into health care professionals' intentions to take actions that address their UB.

The statements are measured using a four-point Likert scale from “strongly disagree” (−2) to “strongly agree” (+2), which was recoded into a binary variable of “disagree” or “agree.” Analysis includes a descriptive summary of each of the measures to include sum, mean, and range followed by bivariate analysis at a significance level of 0.5. A two-sample *t*-test was conducted to test the null hypotheses that there are no differences in either overall IAT D-scores or attitudes toward UB between (1) physicians and nurses, (2) physicians and the general public, or (3) nurses and the general public. Each bivariate analysis also includes an examination by region to detect geographical differences that may be compared to previous research.

## Results

Study respondents were well distributed across each region except the Caribbean, which did not include any physicians and therefore was excluded from further comparative geographical analysis (Table 1). The Frontier had the fewest respondents compared to the Northeast. Physicians and nurses represented ∼3% of the respondents, 1.8% and 2.2%, respectively. Overall, IAT D-scores indicate a slight preference for whites among all respondents (mean, *M*=0.2817, standard deviation, SD=0.44) (Table 2). Health care professionals IAT scores were higher than the general public where nurses showed a slightly greater preference for whites than physicians (0.3331 and 0.3293, respectively) (Table 3).

**Table 1.** Summary of States and Territories Categorized by Region and Occupation

Region	States and Territories	Physicians % (***n***)	Nurses % (***n***)	Other % (***n***)	Total (***N***)
Caribbean	AS, FM, GU, MH, MP, PR, PW, VI	0% (0)	<0% (1)	96% (23)	24
Frontier	AZ, CO, ID, KS, MT, NE, NV, NM, ND, OK, SD, TX, UT, WY	10% (112)	11% (164)	79% (9044)	9320
Northeast	CT, DE, DC, ME, MA, MD, NH, NJ, NY, PA, RI, VT	23% (258)	16% (241)	61% (14,259)	14,758
Midwest	IL, IN, IA, MI, MN, MO, OH, WI	27% (307)	42% (613)	31% (12,891)	13,811
Pacific	AK, CA, HI, OR, WA	17% (192)	17% (249)	66% (11,760)	12,201
South	AL, AR, FL, GA, KY, LA, MS, NC, SC, TN, VA, WV	23% (259)	13% (194)	64% (11,656)	12,109
Total		1.8% (1317)	2.2% (1621)	96% (73,127)	76,065

Physician sample *N*=1317 and Nursing sample *N*=1621; Sampling Frame *N*=76,065; Percentages may not add up to 100% due to rounding.

AL: Alabama; AK: Alaska; AS: American Samoa; AZ: Arizona; AR: Arkansas; CA: California; CO: Colorado; CT: Connecticut; DE: Delaware; DC: District of Columbia; FL: Florida; GA: Georgia; GU: Guam; HI: Hawaii; ID: Idaho; IL: Illinois; IN: Indiana; IA: Iowa; KS: Kansas; KY: Kentucky; LA: Louisiana; ME: Maine; MH: Marshal Islands; MD: Maryland; MA: Massachusetts; MI: Michigan; FM: Micronesia; MN: Minnesota; MS: Mississippi; MO: Missouri; MT: Montana; NE: Nebraska; NM: New Mexico; NV: Nevada; NH: New Hampshire; NJ: New Jersey; NY: New York; NC: North Carolina; ND: North Dakota; MP: Northern Mariana Islands; OH: Ohio; OK: Oklahoma; OR: Oregon; PW: Palau; PA: Pennsylvania; PR: Puerto Rico; RI: Rhode Island; SC: South Carolina; SD: South Dakota; TN: Tennessee; TX: Texas; UT: Utah; VT: Vermont; VI: Virgin Islands; VA: Virginia; WA: Washington; WV: West Virginia; WI: Wisconsin; WY: Wyoming.

**Table 2.** Descriptive Statistics of Race Implicit Association Test D-Scores by Occupation

Occupation	Mean (SD)	Min	Max
Physicians	0.3293 (0.45)	−1.25	1.43
Nurses	0.3331 (0.44)	−1.29	1.47
General public	0.2946 (0.45)	−1.76	1.64

Sampling frame *N*=76,065.

IAT, Implicit Association Test; SD, standard deviation.

**Table 3.** Mean Project Implicit Race Implicit Association Test D-Scores by Occupation and Region

	Nationwide	Pacific	Midwest	Frontier	South	Northeast
Physician	0.3293^a^	0.2763	0.3333^b^	0.3257	0.3437^a,b^	0.3607^a^
Nurses	0.3331^a^	0.2538	0.4039^a^	0.3756^a^	0.2656	0.2976
General public	0.2946	0.2754	0.3089	0.3066	0.2862	0.2947

Indicates a statistically significant difference compared to nurses (b) and the general public (a), *p*-value <0.05.

The majority of respondents tend to agree that the IAT is more an indicator of themselves as individuals, reflecting their automatic thoughts and feelings as opposed to a reflection of their culture which may indicate acknowledgement and acceptance that could lead some respondents to take further action to address their existing biases (Table 4).

**Table 4.** Summary of Attitudes Toward Unconscious Bias by Occupation

My IAT score reflects the culture that I am exposed to, but not me, personally		
Occupation	Disagree	Agree
Physician	47.9% (369)	52.1% (402)
Nurses	36.8% (371)	63.2% (638)
Other	42.2% (17,691)	57.8% (24,213)
Total	42.2% (18,431)	57.8% (25,253)

Whether I like my IAT score or not, it captures something important about me		
Physician	25.9% (201)	74.1% (573)
Nurses	27.2% (275)	72.8% (736)
Other	24.8% (10,477)	75.2% (31,780)
Total	24.9% (10,953)	75.1% (33,089)

The IAT reflects something about my automatic thoughts and feelings concerning this topic		
Physician	26% (201)	74% (572)
Nurses	30.3% (307)	69.7% (706)
Other	26.7% (11,273)	73.3% (30,987)
Total	26.7% (11,781)	73.3% (32,265)

Sampling frame *N*=76,065.

While analysis could find no difference between the overall IAT scores of physicians (*M*=0.329, SD=0.45) and nurses (*M*=0.333, SD=0.43), each was greater than the general public (*M*=0.295, SD=0.45), *p*<0.005. When examined by region, results show that physicians' IAT scores were greater than each of the other groups in the South (*M*=0.3437, SD=0.47) and greater than the general public in the Northeast (*M*=0.3607, SD=0.45). Nurses IAT scores were greater than any other group in the Midwest (*M*=0.4039, SD=0.40) and in the Frontier (*M*=0.3756, SD=0.42).

Upon examining attitudes toward UB, results show that among nurses, there is greater agreement than among any other group that UB is a reflection of one's culture (*M*=0.2658, SD=0.96) and less an indication of individualistic or automatic thoughts toward people of another race (Table 5). Physicians' attitudes (*M*=0.4806, SD=0.87) were more similar to that of the general public (*M*=0.5039, SD=0.86) and reflected the opposite perspective. This observation is consistent across each region except for in the South where agreement among nurses is highest that UB are more the result of individuals' own thoughts and feelings (*M*=0.5206, SD=0.86) than a reflection of the culture (*M*=0.2727, SD=0.96).

**Table 5.** Unconscious Bias Attitudes by Occupation and Region

My IAT score reflects the culture that I am exposed to, but not me, personally						
	Nationwide	Pacific	Midwest	Frontier	South	Northeast
Physician	0.0428^ba^	−0.2126^ba^	0.1160^b^	0^ba^	0.1839	0
Nurses	0.2658^a^	0.2777^a^	0.3209^a^	0.3118	0.2727	0.1141
General public	0.1556	0.1113	0.1962	0.1865	0.1670	0.1311

Whether I like my IAT score or not, it captures something important about me						
Physician	0.4806	0.5905	0.4696	0.5211	0.2686^ba^	0.6058
Nurses	0.4554^a^	0.5104	0.4087^a^	0.4408	0.5372	0.4666
General public	0.5039	0.5363	0.5297	0.4771	0.4711	0.5061

The IAT reflects something about my automatic thoughts and feelings concerning this topic						
Physician	0.4799^b^	0.5312	0.6555^ba^	0.3714	0.1771^ba^	0.6115^ba^
Nurses	0.3932^a^	0.5	0.3410^a^	0.2688^a^	0.5206	0.4324
General public	0.4663	0.4867	0.4852	0.4418	0.4441	0.4665

Values range from disagree (0) to agree (1) and indicates a statistically significant difference compared to nurses (b) and/or the general public (a) or both, *p*-value <0.05.

## Discussion

This study identified that the UB of physicians and nurses and their attitudes toward them differ from the public and in some instances from one another. Health care professionals were found to have a greater preference for whites than the general public. This is contrary to previous work conducted in Colorado, a Frontier state, which found no differences between primary care providers and the general public.^1^ However, a limitation of that study was that it did not examine differences by type of provider and as such was unable to detect the differences identified by this study to support the conclusion made that UB is a societal issue more so than a health care issue.

The findings of this study also demonstrate that in some regions, the UB of nurses show a greater preference for whites, which may be of even greater concern than those of physicians, especially in areas where nurses have full practice authority. Also, preferences toward whites were highest among nurses in the Midwest region. This could be interpreted to suggest that nurses outnumber physicians in this region, indicating that they should be prioritized in research and interventions to address UB in an effort to reduce health care disparities.

However, this study found that nurses' perceived their UB as a reflection of the cultures, to which they are exposed and less as a reflection of their own explicit or unconscious thoughts and feelings regarding race. This may suggest that nurses are less inclined than physicians to participate in UB interventions targeting individuals and are more likely to support those addressing practice and workplace culture. Overall, these observed differences between physicians and nurses may just reflect personal and professional character differences (i.e., elitism, empathy) previously described in the literature.

For example, in a comparison study of empathy among female nurses and physicians, nurses scored higher on 15 out of 20 indicators [64]. Increasing empathy has been described as one method of mitigating UB, which would explain why nurses would have lower IAT scores than physicians.^38–40^

One area of investigation to start would be to examine the contextual factors associated with our health care and medical education systems that may either introduce or reinforce individuals' existing UB. This is supported by previous research demonstrating medical students' false beliefs about race and experiences with racism start in medical school.^8,41–43^

Minimizing the influence of UB to produce disparate health care outcomes necessitates moving beyond individual and interpersonal factors upstream to identify and address systemic issues within education and practice. Emerging literature has begun to describe how medical education and health care are rooted in systemic and oppressive ideologies, such as white patriarchal supremacy, that introduce and/or reinforce students and practitioners explicit and implicit biases, stereotypes, and misbeliefs.^42–46^

Some factors that have already been identified include the poor modeling of patient interactions by faculty, the practice of inferring biological/genetic racial differences in research, and the use of unfounded race correction factors in clinical guidelines. Currently, UB training targeting individuals has been the only tool available to address the downstream effects of systematic racism; however, new resources are emerging in the context of methodologies to address race-based medicine and health care operations using principles of critical race theory.^4,5,38,47–49^

## Conclusion

Overall, these findings suggest that within the health care workforce, the UB of physicians and nurses differ significantly from those of the general public and show significant regional variation. This may explain why UB educational training interventions have not yet demonstrated their effectiveness because the outcome they target is highly variable.^16,50^

More broadly, these findings may also be considered within the context of debates within the social sciences regarding the primacy of individual agency versus structure.^36^ As it relates to health care, the question becomes whether the racially disparate clinical decisions of providers are the result of their own individual autonomy, unconscious or otherwise, or socialization within a system of health care with norms, customs, policies, and so on, designed from its inception to marginalize and minimize the health care needs of racial minorities.

The later further justifies the need to develop and implement interventions that focus on health care systems and culture instead of individuals to reduce health care disparities effectively and sustainably. Other more influential factors external to the individual, such as those associated with education, training, the workplace, and/or community may introduce and/or potentiate UB in a way that makes individual oriented interventions ineffective at reducing health care disparities. While there are studies that instead refocus on system and institutional level approaches to reduce health care disparities, additional research is needed to examine UB in an organizational context and its potential impact on the workforce and patients.^33,37,49,51^

These approaches may be especially embraced by the health care workforce, which has consistently emphasized the need for greater organizational responsibility and accountability to address disparities and other systemic level issues in health care such as burnout and administrative burden.^3,52–54^ Furthermore, the following implications should be considered.

### Policy and education

Several State and Federal policy actions supporting widespread implementation of UB education and training interventions have been proposed and approved.^55,56^ As these findings have demonstrated, at least within the health care workforce, UB is highly variable, to the extent that current educational training interventions may not yet be designed to address. As such, future legislative actions should consider placing a greater emphasis on continued research as opposed to mandates for an intervention that has not yet been proven efficacious nor meets the necessary standards to be considered evidence-based.

Furthermore, continuing medical education mandates are often controversial, do not always result in practice changes and in some instances can create barriers to licensure and certification.^57,58^ However, there still exist the need to create an overall sense of awareness and acknowledgment of the potential impact of UB to influence disparate outcomes, which is the responsibility of those organizations who oversee medical education and training. As suggested, organizations may have some influence on physicians' decisions to address their UB.

## Author Discloser Statement

No competing financial interests exist.

## Abbreviations Used

IAT: Implicit Association Test
SD: standard deviation
UB: unconscious bias
